# Quality improvement strategy to enhance compliance with the World Health Organization Surgical Safety Checklist in a large hospital: Quality improvement study

**DOI:** 10.1016/j.amsu.2020.04.027

**Published:** 2020-05-11

**Authors:** Vania Röhsig, Rubia Natasha Maestri, Mohamed Fayeq Parrini Mutlaq, Aline Brenner de Souza, Artur Seabra, Eliane Reus Farias, Elisiane Lorenzini

**Affiliations:** a CNO Hospital Moinhos de Vento, Porto Alegre, Rio Grande do Sul, Brazil; b Nurse Director Hospital Moinhos de Vento, Porto Alegre, Rio Grande do Sul, Brazil; c CEO Hospital Moinhos de Vento, Porto Alegre, Rio Grande do Sul, Brazil; d Nurse Manager Hospital Moinhos de Vento, Porto Alegre, Rio Grande do Sul, Brazil; e Chief Surgery Center Surgeon Hospital Moinhos de Vento, Porto Alegre, Rio Grande do Sul, Brazil; f Surgery Center Nurse Manager Hospital Moinhos de Vento, Porto Alegre, Rio Grande do Sul, Brazil; g Federal University of Santa Catarina, Florianópolis, Santa Catarina, Brazil

**Keywords:** Surgical safety checklist, Patient safety, Health care quality assurance, Checklist

## Abstract

**Background:**

The World Health Organization Surgical Safety Checklist is an effective tool to reduce morbidity, mortality, perioperative complications, and hospital length of stay. However, its implementation that involves complex social interaction is still challenging.

**Objectives:**

The aim was to increase use of the Surgical Safety Checklist to 100% of performed surgeries compared to current practice at Hospital Moinhos de Vento, in Porto Alegre, Brazil.

**Methods:**

A quality improvement strategy was implemented based on the Plan, Do, Study, Act cycle. During the intervention, Surgical Safety Checklist structure and content were adjusted to the local context and surgeons were engaged in discussions of the medical and scientific basis of the Surgical Safety Checklist. Also, the surgery center nursing team was trained as well as empowered to use the Surgical Safety Checklist.

**Results:**

As compared to baseline data, there was an increase in the use of the tool and data was monitored to evaluate sustainability of the strategy over 26 months. Mean compliance with the Surgical Safety Checklist after the intervention reached 89%. Compliance with the most critical phase – time out – began at 26%. After the intervention, an increase in time out compliance was noted, varying from 60% to 90%.

**Conclusion:**

The proposed quality improvement strategy, implemented at no additional cost to the institution, was effective to increase Surgical Safety Checklist compliance and produced sustainable results.

## Highlights

• It is the first article of this kind in Brazil. This paper describes the implementation of a quality improvement strategy based on the Plan, Do, Study, Act (PDSA) cycle. The aim was to increase use of the Surgical Safety Checklist to 100% of performed surgeries compared to current practice at Hospital Moinhos de Vento, in Porto Alegre, Brazil.
• Our data show that adjustment of World Health Organization Surgical Safety Checklist (WHO SSC) structure and content to the local context, as well as engagement of decision-makers and risk management staff to promote WHO SSC compliance contributed to the success of the proposed intervention.
• Strong points of our quality improvement strategy include the use of an explicit method, namely the Institute for Healthcare Improvement's Plan-Do-Study-Act model, which facilitated the understanding of the intervention/improvement process and allowed us to explore the method's full potential.

## 1 Problem

The implementation of improvements in the surgical routine to increase compliance to the 19-item checklist - World Health Organization Surgical Safety Checklist (WHO SSC) - remains a challenge: there is no consensus regarding best practices and there are no guidelines available in the literature for this purpose. However, there is agreement concerning the need for intensive training of teams and engagement of staff as an essential measure for the appropriation of WHO SSC by professionals for use in clinical practice.

Hospital Moinhos de Vento (HMV), located in the city of Porto Alegre, southern Brazil, is a large 497-bed institution performing an average of 2,000 surgeries monthly. In the past decades, many strategies to improve internal processes have been implemented at HMV, which in 2002 was the first hospital in the South of Brazil to be accredited by the Joint Commission International. In this context, five years ago HMV adopted the WHO SSC. For each surgical procedure, an electronic WHO SSC form must be completed by the nursing team. Adherence to completion of the electronic form, including all checklist items, reached 100%. However, after some time, it became evident that even though the electronic form was filled, not all WHO SSC phases were correctly or routinely performed. Therefore, the risk management team, in partnership with Surgery Center medical and nursing leaders, performed on-site checks over a two-month period. The results showed use of the WHO SSC in 87% of surgeries in the first month and 79% of the surgeries in the second month – that is, on average, 400 procedures every month were performed without application of this safety enhancement mechanism. In this context, the aim was to increase the use of the WHO SSC to 100% of performed surgeries compared to current practice.

## 2 Background

Health care-related adverse events (AE) are frequent, and approximately 40% occur in operating rooms (OR). Nevertheless, 50% of these events are preventable [1]. To reduce this type of preventable injury, in 2008 the World Health Organization (WHO) proposed the WHO SSC. This tool aims at improving compliance with procedures to be performed preoperatively to minimize the risk of intraoperative AE and postoperative complications. The WHO SSC involves specific steps to be followed in three phases: 1) before anesthesia induction (Sign In); 2) before surgical incision (Time Out); 3) and before the patient leaves the OR (Sign Out) [2].

In the past decade, many studies have confirmed the effectiveness of the WHO SSC to reduce morbidity and mortality, perioperative complications, and length of stay [[3], [4], [5]]. This evidence has contributed to the prompt adoption of this tool by many institutions worldwide. However, the challenges of implementing the WHO SSC, considered to be a complex social intervention [6], have been pointed out and discussed in many studies, including some that question the usefulness of the WHO SSC [7,8]. The difficulties linked to WHO SSC implementation are often associated with institutional scenarios in which the checklist was introduced without adequate supporting strategies. Factors such as top-down implementation of the WHO SSC, lack of intensive team training, and mandatory implementation demanded/imposed by regulatory agencies have all been associated with failure, with no improvement in patient outcomes [9,10]. Many investigations have shown that professionals often do not use the WHO SSC correctly, or simply complete the WHO SSC at the end of the surgical procedure, rather than preoperatively [9,[11], [12], [13]]. Also, despite the existence of a manual and other materials to support WHO SSC implementation, this process is not standardized, and in general formal training of teams is not performed [14,15]. In Brazil, studies have shown that the WHO SSC is used inconsistently, with limited compliance, especially when it comes to the Time Out phase [16,17].

Therefore, the present study examines the role of patient safety infrastructure, organizational leadership involvement, and education and training as necessary aspects for effective implementation of the WHO SSC. As noted, the mere introduction of the WHO SSC as a task, without adequate staff or leadership training, may lead to loss of interest and in some cases to disregard of the tool by the surgical team. At HMV, electronic records showed 100% compliance with the WHO SSC. However, close monitoring at the Surgery Center revealed that teams did not comply with some WHO SSC items, or in some cases ignored the WHO SSC.

## 3 Baseline measurement

**Fig. 1:**
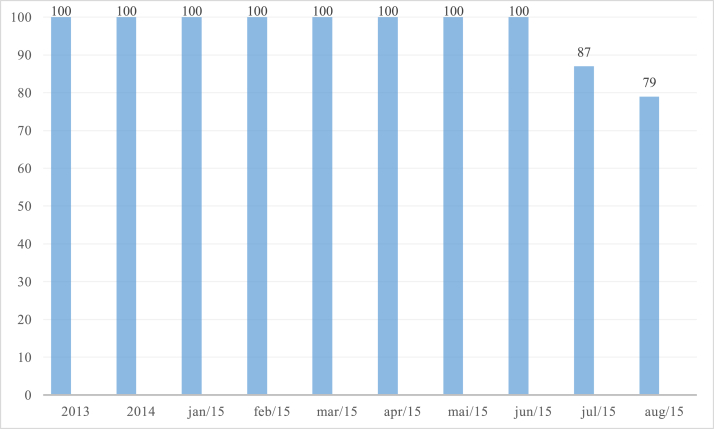
Percent compliance with the Surgical Safety Checklist: data for 2013, 2014, and January–June 2015 were obtained from the HMV information system. July and August 2015 figures were obtained through on-site monitoring.

Data were initially collected from the HMV information system. In addition, compliance data were obtained through on-site monitoring by the risk management team in July and August 2015 (Fig. 1).

## 4 Design

The HMV strategic goals include the continuous improvement of internal processes as to ensure that all activities performed at the hospital are safe. Therefore, actions aimed at improving quality and patient safety are strongly supported and encouraged. In this scenario, the present quality improvement strategy was implemented with the strategic support of the leadership. The planning and execution of activities involved the risk management team, surgeons and nurses, Surgery Center leaders and decision-makers, and Surgery Center teams.

### 4.1 Intervention

The present intervention aimed at engaging surgeons and raising the awareness of teams regarding the need to use the WHO SSC. For that, the WHO SSC was presented as an evidence-based tool with proven effectiveness. That involved discussions about patient risk and real-world safety issues experienced by surgeons. Also, training and education activities were performed, with empowerment of the surgical nursing team to use and, most importantly, to initiate the WHO SSC.

### 4.2 The intervention encompassed

a) Adaptation of WHO SSC structure and content to the local context.
b) Involvement of medical leadership: during the intervention period, the chief Surgery Center surgeon discussed WHO SSC data with physicians from all specialties during routine monthly meetings. The emphasis was on the need for continuous improvement. He also taught a structured evidence-based class showing the effectiveness of the WHO SSC and its very low (virtually zero) cost. As an awareness-raising strategy, he then proposed a comparison between the high cost/limited benefit of developing antineoplastic drugs [18] vs. low cost/recorded impact as high as 30% in improvement of surgical outcomes associated with WHO SSC implementation [1].
c) Sharing of scientific evidence by head surgeons in each specialty: head surgeons acted as multipliers within their specialty teams. They supported the chief surgeon in presenting WHO SSC use indicators at the monthly scientific meetings held by each specialty. They also encouraged open discussions on patient safety during surgery, and mediated experience exchanges and reports of situations involving safety failures, with or without consequences for the patient.
c) Education, training, and empowerment of Surgery Center nursing teams through on-site meetings with risk management staff; standardization of structural and practical aspects relating to the use of the WHO SSC as part of the work flow; encouragement for nursing teams to proactively initiate the WHO SSC Time Out phase immediately before the start of the procedure, directing the pertinent questions to surgeons and anesthetists whenever these physicians did not initiate the WHO SSC themselves; empowerment of nursing team to avoid conflict; nursing team guidance regarding how to report disruptive attitudes and behaviors; resolution of disruptive attitude and behavior cases through face-to-face meetings with chief Surgery Center surgeon.

## 5 Strategy

The aim was to increase the use of the WHO SSC to 100% of performed surgeries compared to current practice. A Plan, Do, Study, Act (PDSA) cycle was implemented. Following implementation of the intervention strategy, as described in Design, WHO SSC compliance was locally audited by a risk management team member. Daily monitoring was performed during 26 months.

**Fig. 2:**
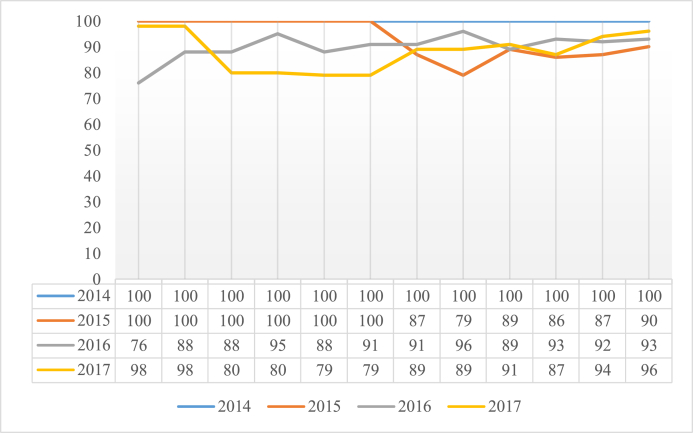
On-site monitoring of WHO SSC use.

The intervention was initially assessed considering the rate of WHO SSC compliance. However, because the aim was to promote learning and continuous improvement, with adjustments in the way the WHO SSC was used, after 12 months the assessment criterion was changed to emphasize to the most critical phase – Time Out. Time Out entails a pause: all activities must stop and team members must focus on the review of relevant details of the procedure that is about to begin. The first month of on-site observation using this assessment criterion revealed 21% compliance, in August 2016 (Fig. 2). The improvement intervention was repeated throughout September.

To increase Time Out compliance, the chief Surgery Center surgeon called new meetings (repetition of intervention) with specialty teams, focusing on scientific evidence supporting use of the WHO SSC. The Surgery Center nursing team and the institution's risk manager organized education and training activities for nursing teams focusing on a detailed examination of the Time Out phase. The requirement of pausing to perform the Time Out step was the main approach. The results show immediate response, with increasing Time Out compliance, as shown in Fig. 2. Data were continuously collected over 14 months for assessment of the effectiveness and sustainability of the intervention.

## 6 Results

Baseline data showed 100% completion of the WHO SSC electronic form. However, on-site checks revealed compliance of 87% in June 2015 and 79% in July 2015. Following the first intervention, in August 2015, increasing compliance was detected, with a steadily ascending curve during the entire period. Mean WHO SSC usage after the intervention was 89%. As described in the Strategy section, all monitoring checks were performed on-site daily during 26 months (Fig. 2).

**Fig. 3:**
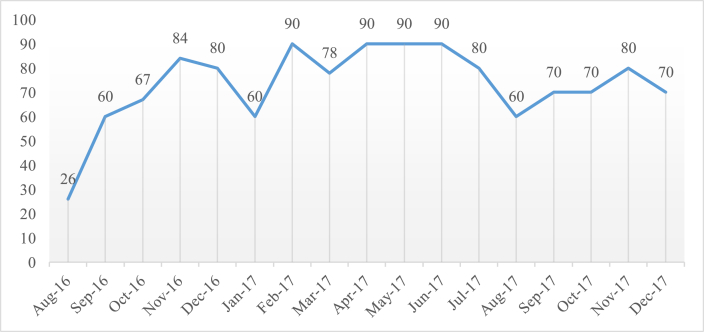
Monitoring of % compliance to WHO SSC Time Out.

In August 2016, the intervention analysis criterion was changed, and the monitoring of Time Out was initiated. The baseline check revealed 26% conformity with the Time Out pause prior to the start of the procedure. After the intervention was repeated, in September 2016, data monitoring revealed an ascending compliance curve. The percent conformity varied from 60% to 90%, showing increased Time Out compliance (Fig. 3).

## 7 Lessons and limitations

The objective of the quality improvement strategy was to achieve 100% WHO SSC compliance compared to current practice. The results do not allow us to infer a direct causal effect of the intervention on WHO SSC use. However, it is possible that the process of engaging and training the Surgery Center team, with involvement of decision-makers, did provide an important contribution to increase WHO SSC adherence, even though the initial goal of 100% was not met.

Continuous data monitoring, during 26 months, showed a lasting increase in compliance, indicating that the strategy has some degree of sustainability. The success of the quality improvement strategy might be related to the engagement of the entire Surgery Center team (physicians, nurses, and nursing technicians), to the ensuing empowerment of the nursing team, and to the intense participation of the risk management team and of physicians and nursing in leadership roles (decision-makers). This is in accordance with the literature, which shows from frontline feedback and engagement of leaders is essential to develop, implement, and increase adherence to interventions aimed at promoting safety [[19], [20], [21]]. In addition, the strategy of showing scientific evidence that support the use of WHO SSC was also effective to raise awareness among physicians. These results corroborate previous findings showing skepticism among surgeons and anesthetists regarding the available evidence. Interestingly, it has been reported that some believe the available evidence to be inconclusive, and thus they do not support broad implementation of the 10.13039/100004423WHO SSC [6].

A key moment that translated into learning for the entire team was the decision to change the intervention analysis criterion. The objectives consisted in promoting learning, continuous improvement, and improving the manner the WHO SSC was used. Thus, monitoring compliance with the most critical WHO SSC phase, Time Out, was initiated. This assessment showed that, despite the intervention and the continuous learning taking place, compliance with Time Out never reached 100% for all items. This is an important datum that reveals the complexity involved in the expected pause during which safety items are checked right before the start of the procedure. According to the literature, further research is necessary to shed light on the means and tools that will ensure that system-level interventions such as the WHO SSC are effectively integrated into practice rather than being seen as just one among many resources [10,22,23].

In addition, all data during the analysis and monitoring indicate that the strategy employed, which did not entail direct implementation cost, is promising to increase the effective use of the WHO SSC. It should also be noted that the present results cannot be generalized given the great influence of local context and institutional culture on paradigmatic issues relating to patient safety and the WHO SSC. Also, the main limitation of the present strategy must be mentioned – which is the fact that only one on-site verification of the Time Out was performed prior to the start of the second intervention round, which focused on improving Time Out compliance. Nevertheless, repeating, monitoring, and adjusting the analysis criterion was important because, despite increased and sustained WHO SSC compliance in the first 12 months of intervention, it became clear that the most critical step, Time Out, was often skipped. Therefore, sharing our experience may contribute to the development of future interventions or WHO SSC-related research.

Strong points of the quality improvement strategy include the use an explicit method, namely the Institute for Healthcare Improvement's Plan-Do-Study-Act model [24], which facilitated the understanding of the intervention/improvement process and allowed the exploration of the method's full potential. In addition, the change in criterion of analysis was duly documented, as recommended by existing international guidelines [25] on the design, performance, and reporting of improvement projects, which require flexibility especially in the design and implementation phases [26].

## 8 Conclusion

It is the first article of this kind in Brazil. The data show that adjustment of WHO SSC structure and content to the local context, as well as engagement of decision-makers and risk management staff to promote WHO SSC compliance contributed to the success of the proposed intervention. The engagement of surgeons in evidence-based discussions and the education, training, and empowerment of the nursing team represent a promising quality improvement strategy, which was sustainable and increased real-world WHO SSC compliance.

Finally, the present learning and continuous improvement experience may be useful to the global surgical community, which must strive to ensure that the WHO SSC is not an end in itself – a checklist that must be filled and followed to fulfill a task – but rather a part of the care process associated with successful surgical procedures.

## Ethical approval

The project from which we extracted this data was approved by the ethics committee (CAAE: 57679316.9.0000.5330 - approval 1.833.572) at Moinhos de Vento Hospital -Institute of Education and Research.

## Sources of funding

There is no sources of funding.

## Author contribution

**Vania Röhsig, Rubia Natasha Maestri, Mohamed Fayeq Parrini Mutlaq, Aline Brenner de Souza, Elisiane Lorenzini** made substantial contributions to the conception and design of the work, or the acquisition, analysis or interpretation of data, and to drafting the work or revising it critically for important intellectual content. They made a final approval of the version published and agree to be accountable for all aspects of the work in ensuring that questions related to the accuracy or integrity of any part of the work are appropriately investigated and resolved.

**Artur Seabra and Eliane Reus Farias** made substantial contributions to the acquisition of the data and to drafting the work. They approved the final version and agree to be accountable for all aspects of the work in ensuring that questions related to the accuracy or integrity of any part of the work are appropriately investigated and resolved.

## Trial registry number

None.

## Guarantor

Elisiane Lorenzini.

## Provenance and peer review

Not commissioned, externally peer reviewed.

## Ethical considerations

Data for this report were collected from the HMV information system. The research project from which we extracted this data was approved by the ethics committee (CAAE: 57679316.9.0000.5330 - approval 1.833.572) at Moinhos de Vento Hospital - Institute of Education and Research.

## Consent

There is no patient or any people data in this study. No informed consent was used in this study.

## Registration of research studies

Name of the registry: A quality improvement strategy to enhance compliance with the World Health Organization Surgical Safety Checklist in a large hospital: Case report.

Unique Identifying number or registration ID: Research Registry UIN 5497.

Hyperlink to the registration (must be publicly accessible): https://www.researchregistry.com/register-now#home/registrationdetails/5e8f2e7f9b8db6001691c17c/

## Declaration of competing interest

There is no conflicts of interest.
